# Supplementary material for: Lost in the North Sea—Geophysical and geoarchaeological prospection of the Rungholt medieval dyke system (North Frisia, Germany)
Source: PLoS One. 2022 Apr 4;17(4):e0265463. doi: 10.1371/journal.pone.0265463 (PMC8979465; doi:10.1371/journal.pone.0265463)

PLOS ONE

Lost in the North Sea - Geophysical and geoarchaeological prospection of the Rungholt medieval dyke system (North Frisia, Germany)

--Manuscript Draft--

**Manuscript Number:**	PONE-D-21-06483
**Article Type:**	Research Article
**Full Title:**	Lost in the North Sea - Geophysical and geoarchaeological prospection of the Rungholt medieval dyke system (North Frisia, Germany)
**Short Title:**	Tidal flat archaeological prospection
**Corresponding Author:**	Dennis Wilken, Ph.D.<br>University of Kiel: Christian-Albrechts-Universitat zu Kiel<br>Kiel, GERMANY
**Keywords:**	Rungholt; archaeological prospection; geophysics; magnetic gradiometry; seismic; vibracore; geoarchaeology; frisian; medieval
**Abstract:**	We performed geophysical and geoarchaeological investigations in the Wadden Sea off North Frisia (Schleswig-Holstein, Germany) to map the remains and to determine the state of preservation of the medieval settlement of Rungholt, especially its southern dyke segment, called the {\it Niedam} dyke. Based on archaeological finds and historical maps, Rungholt is assumed to be located in the tidal flats around Hallig Südfall, a tidal flat island. During medieval and early modern times, extreme storm events caused major land losses, turning cultivated marshland into tidal flats. Especially the 1st Grote Mandrenke (or St. Marcellus' flood), an extreme storm surge event in 1362 AD, is addressed as the major event that flooded and destroyed most of the Rungholt cultural landscape. Cultural traces like remains of dykes, drainage ditches, tidal gates, dwelling mounds or even plough marks were randomly surveyed and mapped in the tidal flats by several authors at the beginning of the 20th century. Due to the tidal flat dynamics with frequently shifting tidal creeks and sand bars, the distribution of cultural remains visible at the surface is rapidly changing, making it hard to create a comprehensive map of the cultural landscape by surveying. Today, the Niedam dyke area is fully covered by tidal flat sediments, depriving any remains from further archaeological investigation. Since little is known about the precise location or state of preservation of these remains, our investigation aimed at the rediscovery of the medieval dyke system and associated structure with modern and accurate geophysical, geodetical and geoarchaeological methods.<br>Magnetic gradiometry revealed a large part of the medieval dyke, confirming two tidal gates and several terps connected inland with the dyke, providing a detailed example of a Frisian medieval dyke system. Based on our results, the so far inaccurate and incomplete maps of this part of Rungholt can now be specified and completed. Beyond that, seismic reflection profiles give a first depth resolving insight in the remains of the dyke system, revealing a severe threat to the medieval remains by erosion. The site is exemplary for the entire North Frisian coast, that was influenced by multiple flood events in the middle ages to modern times.
**Order of Authors:**	Dennis Wilken, Ph.D.
	Hanna Hadler
	Tina Wunderlich
	Bente Sven Majchzack
	Michaela Schwardt
	Annika Fediuk
	Peter Fischer
	Timo Willershäuser
	Stefanie Klooß
	Andreas Vött
	Wolfgang Rabbel

Powered by Editorial Manager® and ProduXion Manager® from Aries Systems Corporation

Additional Information:	
**Question**	**Response**
**Financial Disclosure**<br><br>Enter a financial disclosure statement that describes the sources of funding for the work included in this submission. Review the submission guidelines for detailed requirements. View published research articles from *PLOS ONE* for specific examples.<br><br>This statement is required for submission and **will appear in the published article** if the submission is accepted. Please make sure it is accurate.<br><br>**Unfunded studies**<br>Enter: *The author(s) received no specific funding for this work.*<br><br>**Funded studies**<br>Enter a statement with the following details:<br>• Initials of the authors who received each award<br>• Grant numbers awarded to each author<br>• The full name of each funder<br>• URL of each funder website<br>• Did the sponsors or funders play any role in the study design, data collection and analysis, decision to publish, or preparation of the manuscript?<br>• **NO** - Include this sentence at the end of your statement: *The funders had no role in study design, data collection and analysis, decision to publish, or preparation of the manuscript.*<br>• **YES** - Specify the role(s) played.<br><br>* typeset	This study was carried out within the framework of the priority program 1630 -Harbours from the Roman Periods to the Middle Ages- by the German Research Foundation (RA 496/26-2, ZI 721/10-2, 20/7-2, HA 7647/1-1, VO 938/21-1) and the ROOTS Cluster of Excellence funded by<br>the German Research Foundation (EXC 2150-390870439).<br>The funders had no role in study design, data collection and analysis, decision to publish, or preparation of the manuscript.
**Competing Interests**<br><br>Use the instructions below to enter a competing interest statement for this submission. On behalf of all authors, disclose any competing interests that could be perceived to bias this work—acknowledging all financial support and any other relevant financial or non-financial competing interests.	The authors have declared that no competing interests exist.

This statement is required for submission and **will appear in the published article** if the submission is accepted. Please make sure it is accurate and that any funding sources listed in your Funding Information later in the submission form are also declared in your Financial Disclosure statement.<br><br>View published research articles from *PLOS ONE* for specific examples.<br><br>**NO authors have competing interests**<br><br>Enter: *The authors have declared that no competing interests exist.*<br><br>**Authors with competing interests**<br><br>Enter competing interest details beginning with this statement:<br><br>*I have read the journal's policy and the authors of this manuscript have the following competing interests: [insert competing interests here]*<br><br>* typeset	
**Ethics Statement**<br><br>Enter an ethics statement for this submission. This statement is required if the study involved:<br><br>• Human participants<br>• Human specimens or tissue<br>• Vertebrate animals or cephalopods<br>• Vertebrate embryos or tissues<br>• Field research<br><br>Write "N/A" if the submission does not require an ethics statement.<br><br>General guidance is provided below. Consult the submission guidelines for detailed instructions. **Make sure that all information entered here is included in the**	N/A

**Methods section of the manuscript.**<br><br>**Format for specific study types**<br><br>**Human Subject Research (involving human participants and/or tissue)**<br>• Give the name of the institutional review board or ethics committee that approved the study<br>• Include the approval number and/or a statement indicating approval of this research<br>• Indicate the form of consent obtained (written/oral) or the reason that consent was not obtained (e.g. the data were analyzed anonymously)<br><br>**Animal Research (involving vertebrate animals, embryos or tissues)**<br>• Provide the name of the Institutional Animal Care and Use Committee (IACUC) or other relevant ethics board that reviewed the study protocol, and indicate whether they approved this research or granted a formal waiver of ethical approval<br>• Include an approval number if one was obtained<br>• If the study involved *non-human primates*, add *additional details* about animal welfare and steps taken to ameliorate suffering<br>• If anesthesia, euthanasia, or any kind of animal sacrifice is part of the study, include briefly which substances and/or methods were applied<br><br>**Field Research**<br><br>Include the following details if this study involves the collection of plant, animal, or other materials from a natural setting:<br>• Field permit number<br>• Name of the institution or relevant body that granted permission	
**Data Availability**<br><br>Authors are required to make all data underlying the findings described fully available, without restriction, and from the time of publication. PLOS allows rare exceptions to address legal and ethical concerns. See the PLOS Data Policy and FAQ for detailed information.	Yes - all data are fully available without restriction

Powered by Editorial Manager® and ProduXion Manager® from Aries Systems Corporation

A Data Availability Statement describing where the data can be found is required at submission. Your answers to this question constitute the Data Availability Statement and **will be published in the article**, if accepted.<br><br>**Important:** Stating 'data available on request from the author' is not sufficient. If your data are only available upon request, select 'No' for the first question and explain your exceptional situation in the text box.<br><br>Do the authors confirm that all data underlying the findings described in their manuscript are fully available without restriction?	
**Describe where the data may be found in full sentences. If you are copying our sample text, replace any instances of XXX with the appropriate details.**<br><br>• If the data are **held or will be held in a public repository**, include URLs, accession numbers or DOIs. If this information will only be available after acceptance, indicate this by ticking the box below. For example: *All XXX files are available from the XXX database (accession number(s) XXX, XXX.*).<br>• If the data are all contained **within the manuscript and/or Supporting Information files**, enter the following: *All relevant data are within the manuscript and its Supporting Information files.*<br>• If neither of these applies but you are able to provide **details of access elsewhere**, with or without limitations, please do so. For example:<br><br>*Data cannot be shared publicly because of [XXX]. Data are available from the XXX Institutional Data Access / Ethics Committee (contact via XXX) for researchers who meet the criteria for access to confidential data.*<br><br>*The data underlying the results*	All core stratigraphies are made available to the Schleswig-Holstein State Geological archives. Archaeological find data are recorded by the State Archaeological office and integrated into the Schleswig-Holstein Archaeological Database (ADSH). Research data are entered into the web-based information system of the cultural landscape cadastre KuLaDig (www.KuLaDig.de, Ickerodt 2017) introduced in Schleswig-Holstein and coordinated by the Rhineland Regional Council and made available to a broad public. All basic data as well as new results are entered into a joint GIS project that is accessible via a central project server of Johannes-Guttenberg-University Mainz in cooperation with the Center for Data Processing at JGU.

Powered by Editorial Manager® and ProduXion Manager® from Aries Systems Corporation

presented in the study are available from (include the name of the third party and contact information or URL).<br>• This text is appropriate if the data are owned by a third party and authors do not have permission to share the data.<br><br>* typeset	
Additional data availability information:	Tick here if the URLs/accession numbers/DOIs will be available only after acceptance of the manuscript for publication so that we can ensure their inclusion before publication.

Powered by Editorial Manager® and ProduXion Manager® from Aries Systems Corporation

Manuscript
Click here to access/download;Manuscript;Rungholt.pdf

Lost in the North Sea - Geophysical and geoarchaeological prospection of the Rungholt medieval dyke system (North Frisia, Germany)

Dennis Wilken[1], Hanna Hadler[2], Tina Wunderlich[1], Bente Majchczack[3], Michaela Schwardt[1], Annika Fediuk[1], Peter Fischer[2], Timo Willershäuser[2], Stefanie Klooß[4], Andreas Vött[2], Wolfgang Rabbel[2],

1 Institute of Geosciences, Kiel University, Kiel, Germany
2 Institute for Geography, Johannes Gutenberg-University Mainz, Mainz, Germany
3 Cluster of Excellence ROOTS, Kiel University, Kiel, Germany
4 Archaeological State Department Schleswig-Holstein, Germany

∗ E-mail:dennis.wilken@ifg.uni-kiel.de

Abstract

We performed geophysical and geoarchaeological investigations in the Wadden Sea off North Frisia (Schleswig-Holstein, Germany) to map the remains and to determine the state of preservation of the medieval settlement of Rungholt, especially its southern dyke segment, called the *Niedam* dyke. Based on archaeological finds and historical maps, Rungholt is assumed to be located in the tidal flats around Hallig Südfall, a tidal flat island. During medieval and early modern times, extreme storm events caused major land losses, turning cultivated marshland into tidal flats. Especially the 1st Grote Mandrenke (or St. Marcellus' flood), an extreme storm surge event in 1362 AD, is addressed as the major event that flooded and destroyed most of the Rungholt cultural landscape. Cultural traces like remains of dykes, drainage ditches, tidal gates, dwelling mounds or even plough marks were randomly surveyed and mapped in the tidal flats by several authors at the beginning of the 20th century. Due to the tidal flat dynamics with frequently shifting tidal creeks and sand bars, the distribution of cultural remains visible at the surface is rapidly changing, making it hard to create a comprehensive map of the cultural landscape by surveying. Today, the Niedam dyke area is fully covered by tidal flat sediments, depriving any remains from further archaeological investigation. Since little is known about the precise location or state of preservation of these remains, our investigation aimed at the rediscovery of the medieval dyke system and associated structure with modern and accurate geophysical, geodetical and geoarchaeological methods. Magnetic gradiometry revealed a large part of the medieval dyke, confirming two tidal gates and several terps connected inland with the dyke, providing a detailed example of a Frisian medieval dyke system. Based on our results, the so far inaccurate and incomplete maps of this part of Rungholt can now be specified and completed. Beyond that, seismic reflection profiles give a first depth resolving insight in the remains of the dyke system, revealing a severe threat to the medieval remains by erosion. The site is exemplary for the entire North Frisian coast, that was influenced by multiple flood events in the middle ages to modern times.

Introduction

The wadden region along the North Sea coast is an area of dyked or formerly dyked salt marshes and reclaimed coastal peat bogs (Vollmer et al., 2001). This coastal wetland contains visible and past human adaptations to the environment in the form of embankments, dykes, canals, polders, making it a cultural landscape of exceptional cultural historical value (Vollmer et al., 2001; Bazelmans et al., 2012). Natural and human-influenced dynamics have changed the marshes and tidal flats throughout time. These changes are especially visible by the numerous traces of medieval and early modern settlements and remains of their cultural landscapes that appear and disappear in the ever-changing environment of the Wadden Sea. In the Rungholt area, a medieval settlement in North Frisia (Germany), most archaeological remains date to the 12th to 14th cent. AD, a period when a wave of immigrating Frisian settlers extensively cultivated the wide coastal marshes and fenlands. Today, only little is known about the appearance of this medieval landscape. During the Medieval and Early Modern Periods, major storm events in 1362, 1634 and 1717 significantly re-shaped the North Frisian coastal area, destroying wide parts of the cultural landscape and turning them into the tidal flats of the present-day Wadden Sea. During the 1362 AD event, the coastline in the study area abruptly retreated about 25 km inland, with only minor patches of marshland left in between (Newig, 2014). The part of Rungholt, that is investigated in

Fig 1. Study site map a) Location of the study area at the North Sea coast of Germany. Grey areas show the tidal flats and yellow areas show the recent coastline. The dashed line indicates an approximate model of the former coastline of the island *Strand* before the Marcellus flood 1362 AD. b) Aerial photo of the tidal island *Hallig Südfall* and surrounding tidal flats (LVermLKN, 2018). The white transparent box indicates the investigated site, named *Niedam area*, dots mark known archaeological findings recorded in the catalogue of the archaeological state department of Schleswig-Holstein.

the present study, (Fig. 1) is well known for its high density of cultural remains: groups of dwelling mounds, a wide network of drainage ditches, large dykes and the remains of two wooden tidal gates (Kühn, 2007; Hadler et al., 2018b) were first observed by Busch (1923; 1963), when areas of medieval marshland were exposed after erosion of parts of the overlying, geologically younger tidal island of Südfall (Bantelmann, 1966). These remains are assumed to be a central part of the Rungholt dyke system. Rungholt was known from historical tradition to have been lost in the flood of 1362 AD (e.g. Busch, 1923; Meier, 2013; Panten, 2012; 2016a; 2016b). Archaeological finds from the tidal flats around Hallig Südfall indicate the importance of the medieval settlement. They include imported goods from the Rhineland, Flanders and even Spain, namely pottery, metal vessels, metal ornaments and weapons (Kühn, 2016). West and southwest of Südfall, two distinctive settlement areas can be distinguished: the so called *8-Warften area* and the *Niedam area*, whereas the latter was decribed by Busch (1923; 1964; 1971) as a possible harbour site, comprising features typical of a tidal-gate associated harbour (so-called "Sielhafen", Barmeyer (1975)). Fig. 2 a) maps the significant structures that were visible at the surface at the beginning of the 20th century as described by Busch (1923; 1971) and others (e.g. Muuß, 1934). Fig. 2 b) sketches the features described in terms of the dyke systems. In the western part of the investigated dyke section, remains of two wooden tidal gates were described - dating to the 12th to 14th cent. AD (Hadler et al., 2018a). The inner chambers have a length of c. 25 m, indicating a dyke base of 25 m to 30 m. So far, contemporary gates of similar size are only known from the medieval harbour of Rotterdam (Carmiggelt & Guiran, 2002). On the landward side,

rectangular dwelling mounds are directly attached to the dyke and provide sufficient space for settlement and trading activites (i.e. houses, storage facilities). Detached dwelling mounds mainly belong to farmsteads.

Fig 2. Findings and dyke model. a) map summarizing all significant archaeological structures that were visible in the tidal flats and recorded by a local resident during the early 20th century (Busch, 1923; 1971). Observations were usually located by bearing a fix object on the mainland or the island and triangulation (Busch, 1971). b) sketch illustrating the main features of the coastal environment connected to the medieval dyke. Typical rectangular dwelling mounds were either attached to the dyke or lay isolated in drained cultivated marshlands and protected the settlements from being flooded. The report by Busch (1923; 1963) further shows two tidal gates embedded in the dyke, that opened during low tide to drain the marshes on the landward side but closed with the incoming tide.

With the presented geoarchaeological and geophysical prospection approach we aim first and foremost at determining the exact location and extent of the cultural remains connected to the dyke system in the Niedam area, as they are described and mapped in the older research tradition (e.g. Busch, 1971). By using modern prospection techniques including high resolution depth imaging methods, we aim at a comprehensive picture of the archaeological features; advancing from repeated, random surveying and sighting in the past towards a systematic prospection. Based on the prospection data, we seek to re-interpret the Niedam-dyke settlement and harbour area. Furthermore, we will explore to which extent the archaeological structures are preserved after several decades of erosion, flooding events and ongoing sediment transport due to the ever-changing tidal flat morphology since their original discovery in the 1920s. This leads to the question, to which degree the archaeological features beneath the tidal flats have been eroded and how pressing the need for further exploration in the tidal flats is.

0.1 History of land use and reclamation in the Rungholt area

The preconditions for medieval settlement dynamics largely depended on the palaeogeographic evolution of the North Frisian area during the Holocene. In the course of the post-glacial sea level rise, sand-spits formed west of today's Wadden Sea, closing the area off from a direct marine influence and leading to the formation of a quiescent brackish lagoonal system repeatedly affected by extensive peat formation (Bantelmann, 1966; Hoffmann, 1988). Since 2000 BC, ongoing siltation has formed wide fenlands and marshes, traversed by different types of waterbodies. In the mid-1st millennium BC, the marine influence is almost negligible (Hoffmann, 2004). The fenlands were first used by man in the late Neolithic and early Bronze Age (Harck, 1980; Bantelmann, 1966), but it was not before the 8th cent. AD that a first group of Frisian immigrants settled in favorable, elevated coastal marshes on unprotected level-ground settlements (Kühn, 2007) during a time of stagnation in sea-level rise in the 8th-10th century (Behre, 2007; Bungenstock and Weertz, 2010). It appears, that from the 11th century AD onward, an increased marine influence endangered the low marshlands of North Frisia by flooding events (Bantelmann, 1966; Hoffmann, 1988; 2004; Kühn/Müller-Wille, 1988; Menke, 1988). Immediate reactions to protect settlements from flooding were the construction of artificial dwelling mounds or terps. At the same time, the settlers sought to reclaim larger areas of marsh- and fenlands for agriculture, which had to be protected from floods. Thus, first dykes, constructed in the late 11th century AD, were either built as ring dykes around farmland or between dwelling mounds, enclosing land for farming and settlements (Kühn, 1992a; 1992b; 2007; Bazelman et al. 2012) and forming polders (=

koog or groden). Archaeological observations on high medieval dykes and dwelling mounds showed that contemporary dykes reached only heights of up to 1.6 m to 2.0 m above sea-level. Therefore, it has been argued that the primary function of the early dykes was to protect the farmland against seasonal flooding during the summer and only the terps were sufficiently protected against major storm surges (Kühn, 1992a; Kühn/Müller-Wille, 1988; Meier, 2013). While the early dyking efforts were confined to small and dispersed areas, large-scale land reclamation of the 12th-13th century required construction of long coastal dykes to enclose and protect large areas. Consequently, the dyked inland had to be drained through tidal gates that could at the same time serve as landing sites or harbours (Barmeyer, 1975; Hadler et al., 2018b). The know-how needed for dyke construction, fenland-cultivation and water-management was probably brought by a second wave of Frisian immigrants from the southern North Sea coast (Århammar, 1995; Kühn, 1992a; 2007). Peat extraction for soil melioration, firing and salt production as well as intense draining of marshland caused subsidence of the ground surface, increasing the potential for flooding and permanent land losses after breaching of dykes as it is recorded for the major flooding events in 1362 AD and later (Hadler et al., 2018a; Hoffmann, 1988; Meier, 2004).

Methods

The tidal flat environment of the study area (Fig. 1b) bears several difficulties for performing geophysical and geoarchaeological prospection. First, there is a very narrow time window whilst the area is accessible by walking during low tide. This leads to the second limitation which is the general accessibility. The main investigation area lies about one kilometer off Hallig Südfall. This distance has to be considered and passed during the low tide time window, leaving about two hours of measuring time for one low tide phase. Furthermore, accommodation is neither available nor allowed on the Hallig, making the prospection team dependant on daily transfer from the mainland to the island (about 7 km). During high tide, boat based measurements can be performed, starting at a jetty in the south of Südfall. Measurement time is slightly longer than two hours, and is only reduced by the water depth (about 1 m) at which the boat is able to leave and return to the island. This leads to the following available datasets in the tidal flat area around the southern part of the Rungholt dyke system, that were collected in two field campaigns in 2016 and 2017 in the framework of the DFG Priority Programme SPP 1630: 'Harbours from the Roman Period to the Middle Ages' (von Carnap[U+2010]Bornheim & Kalmring, 2011):

1. **Magnetic gradiometry** data, collected two hours each day during good weather conditions and good sight. The data can be acquired on tidal flat areas that fall dry during low tide. The area that is accessible by walking with a magnetic cart is mainly made of sandbars with only a small portion of mud.

2. **Marine reflection seismic** data, collected about two to three hours a day during high tide and calm sea conditions. Wave height should be less than half a meter to avoid wave motion noise and image distortion due to tilted source and receivers.

3. **Vibracore samples** and subsequent laboratory analysis during low tide.

4. Some archaeological features become visible time by time due to erosion of the tidal flats. Thus archived **aerial photos** can show archaeological features that are no more visible today, thereby improving parts of the model in Fig. 2 a).

Magnetic Gradiometry

Magnetic gradiometry measures the vertical difference of the vertical component of the magnetic field of the Earth. The difference is independent of regional fields and therefore mainly measures the local magnetic field caused by bodies in the shallow part of the ground. A magnetic gradiometer survey was performed using an array of six fluxgate gradiometers (Foerster fluxgate differential vertical component magnetometers) with an internal vertical sensor distance of 0.65 m, a horizontal sensor spacing of 0.5 m and a sampling frequency of 20 Hz mounted on a cart especially built for the tidal flat environment (Fig. 3 a). Accurate positioning was achieved by RTK (Real Time Kinematic) DGPS (Leica 530). The arithmetic mean was subtracted from the data of each profile in order to eliminate the constant portion of the magnetic field caused by instruments installed on the instrument vehicle. Noisy profiles are removed and some profiles are high pass filtered with a cutoff of k=0.002 1/sample if they still showed a long wavelength trend after mean substraction. All remaining profiles are low-pass filtered with a cutoff wavenumber of k=0.06 1/sample to remove short wavelength noise such as walking or movement noise. The resulting values were binned inline and interpolated cross-line between neighbouring profiles in order to form a data grid of 0.2 m bin size. Finally the area is kk-filtered (Wilken et al., 2012) using a pieslice filter operator of 20° opening, cosine tapered at the edges, to remove remaining stripe noise from the data.

Fig 3. Methods a) Magnetic cart built for prospection in tidal flat areas. Large wheels prevent the system to get stuck in muddy areas of the tidal flats. b) The used marine seismic acquisition system mounted on an inflatable catamaran in front of a small rubber dinghi. The equipment is made for lightweight transport to the island and based on the system used in Wilken et al. (2019b). c) Vibracoring in the tidal flat area. Equipment needs to be carried by special carts (seen in the front) d) Map showing the part of the study area that was accessible to magnetics and vibracoring, the position of the corings, and the seismic lines recorded during the two campaigns.

Marine reflection seismics

We used a high resolution two channel seismic reflection (sediment echosounder) system, and RTK-DGPS positioning mounted on an inflatable catamaran (Fig. 3 b). Comparable prospection setups have been used by Westley and Mcneary (2017), Quinn et al. (2002), Gron and Boldreel (2013), Wilken et al. (2019a, 2019b). The seismic acquisition system consisted of a piezoelectric transducer (ELAC Nautik TL-444, 4 kHz center frequency) that acts as seismic source, and two hydrophones. The transducer is driven with a Fuchs-Müller wavelet of 4 kHz center frequency. After passing the transfer functions of source and hydrophone transducer, the system creates a signal with a bandwidth from 2 kHz to 6 kHz and a peak frequency of 3.5 kHz. Taking 3.5 kHz as resulting peak frequency and 1480 m/s as wave velocity in water, the theoretical vertical resolution is about 0.1 m. The horizontal resolution is defined by the first Fresnel zone, which depends on the depth of the reflector and ranges from 0.45 m (0.5 m depth) to 3.5 m (30 m depth). However, as we were not aiming at imaging small scale objects but at imaging the stratigraphy of mainly 2D structures (dyke) or structures with a horizontal extent of several meters (terps), the horizontal resolution is not as important as the vertical. Further details about the used transducer and logger technique can be found in Wilken et al. (2019a). The horizontal data sampling depends on the signal repetition rate (8 Hz) and travel speed of the boat, which led to an average spacing of

about 0.16 m between data points along the boat track. Time data sampling frequency was set to 35.7 kHz with a record length of 57 ms. The positioning is performed by a Leica 1200 RTK-DGPS. Data processing included the following steps:

- Bandpass filtering using a Butterworth filter opening at 1 kHz to 2 kHz and closing from 6 kHz to 7 kHz
- Deconvolution using a fixed filter operator, derived from Wiener predictive error deconvolution of an isolated seafloor reflection signal in deeper water, which is convolved with the full seismic trace.
- Automatic picking of the seafloor reflection and smoothing with a moving average of 120 traces to suppress wave motion, and removal of the seafloor reflection.
- Semblance-based coherence filter (Milkereit and Spencer, 1989).
- Geometrical spreading correction using a linear time-gain function.
- Migration of the data using Stolt migration (Stolt, 1978) with a constant velocity of 1480 m/s.

Vibracoring

Vibracores were drilled at selected locations using an engine-driven coring device (type Atlas Copco Cobra pro) and closed steel augers with plastic liners of 5 cm diameter in order to calibrate geophysical prospection results. The maximum coring depth reached 4 m b.s. (below surface). All cores were opened, cleaned, photographed, described, and sampled in the laboratory. Descriptions of stratigraphic units followed the standard procedure given by Eckelmann (2005) and Schrott (2015) and comprised criteria like grain-size, sediment colour, carbonate content, macrofossil content, archaeological artefacts, etc. Sedimentary logs were created using the GGU-STRATIG software (Civilserve GmbH, Steinfeld, Germany). Analyses of sedimentary, geochemical and microfaunal paleoenvironmental parameters (e.g. grain size, loss on ignition, element concentrations, magnetic susceptibility, foraminifers, and ostracods) allowed to identify different sedimentary environments. For a detailed description of the approach, see Hadler et al. (submitted).

The geochronological framework is based on dendrochronological dating of a wooden beam from the larger tidal gate (published in Hadler et al. (2018b)) and archaeological age estimations of finds from the tidal flats (Kühn, 2016).

Results

Magnetic Gradiometry

Fig. 4 a) shows the magnetic map of the investigated area. In general the amplitude of anomalies in the area show a very small dynamic of about 2 nT. Several features can be observed in the map and are shown in fig. 4 b) as a redrawing of the visible anomalies. First of all, the map is divided in three main areas (roman numbers I, II, and III), which show a general offset in magnetic signal amplitude in comparison to each other, although single features can be observed continuously passing through all areas. Area I is the area of least magnetic signal amplitude, whereas area III shows a generally increased, bumpy signal. Beside this separation, several local features were observed and labeled as follows:

1. Feature IV: an elongated structure passing the whole area. In comparison to Figure 2 (red, dotted rectangle), and based on its shape, this can directly be connected to the dyke.

2. Feature V: several nearly rectangular features that directly connect to the inside of the dyke.

3. Feature VI: elongated structures that intersect feature II

4. Feature VII: two linear anomalies beginning at the position of the tidal gates (based on 2) and going south, then being connected to a faint area of negative anomaly.

5. Feature VIII: elongated accumulations of small dipoles

6. Feature IX: weak anomaly north of the expected positions of the tidal gates.

Fig 4. Results magnetic gradiometry a) Magnetic map of the tidal gate and western dyke covering the part of the investigation area that was accessible by walking. b) Redrawing of the magnetic map based on a comparison with the recorded observations in Fig. 2a. I, area of reduced magnetic signal amplitude compared to the areas II and III; IV, elongated structure, related to the dyke; V, signatures that are connected to the inner side of the dyke; VII, anomalies connected to the tidal gate position that is probably related to I; VI elongated anomalies with no corresponding structures in the recorded observations.

Vibracoring

Five vibracores were drilled at representative sites to identify and calibrate the different types of anomalies encountered by magnetic gradiometry and reflection seismics. Regarding the magnetic map, vibracores RUN 13A (-0.65 m b.s.) and RUN 25A (-0.85 m b.s.) were drilled in areas I and II to the south of the elongate structure of feature IV. RUN 20A (-0.92 m b.s.) and RUN 26A (-0.69 m b.s.) were drilled approx. 200 m apart in feature IV, while RUN 27A (-0.65 m b.s.) lies just north of structure VI at the outer margin of a rectangular feature V. Following Hadler et al. (2021), seven different facies types were identified from vibracore stratigraphies (Fig. 5a). Facies type description follows from bottom to top; labels are according to Hadler et al. (2021). Facies type F makes up the lowermost part of all the vibracores and consist of grey to dark grey silty sediments. Deposits reflect low-energy depositional conditions of a brackish to marine shallow water quiet reach environment with predominantly anoxic/reducing conditions sheltered from wave dynamics and/or currents.

Fig 5. Results vibracore transects a) Vibracore locations and stratigraphies in relation to magnetic prospection results. b) Transect A shows the increasing thickness of recent tidal flat deposits (facies type H) in northern direction. c) Transect B shows the limited occurrence and different depth levels of facies type D in the eastern part of the study site.

Facies type G consist of layers of sand or shell debris that occasionally intersect facies type F. Deposits reflect sporadic increases of wave dynamics and/or currents, e.g. by strong storm surges or shifting tidal inlets.

Facies type E shows light grey silty sediments that are penetrated by thick roots of reed. Deposits reflect a distinct change from brackish-marine conditions of facies type F to shallow water pioneer zone conditions, characterized by increased salinity (fluctuations) and accumulation of organic-rich mud (Reineck, 1982). Microbial reduction of sea water sulphides is known to significantly enrich S in freshly deposited muds (Schroeder and Brümmer, 1968) and a distinct mottling along the upper boundary indicates the development of a characteristic mono- and disulphide zonation (Reineck, 1982). Facies type E was only encountered in vibracores RUN 13A, RUN 26A and RUN 27A.

Facies type D2 is made of homogeneous mud of light grey colour, indicating a reductive environment. Palaeoenvironmental proxies reflect semi-terrestrial conditions of a frequently flooded pioneer zone to lower salt marsh environment, where constant siltation rises the ground surface to some decimetres below MHW, inducing environmental stress by high fluctuations in salinity and temperature during tides and/or seasons (Reineck, 1982; Beeftink & Rozema, 1993) as well as initial soil formation processes (Scheffer & Schachtschabel, 2018).

Facies type D1 consists of compact, bluish grey clayey mud with strong hydromorphic features. The deposit shows clear characteristics of a high salt marsh environment, where ongoing sedimentation of fine-grained, organic-rich sediments raised the ground level above MHW, intensifying desalinization and soil aeration (Bartholdy, 1997; 2012). Subsequent oxidation of (Fe-)sulphides and the decay of organic matter produce Fe-oxides, sulphuric and carbonic acid, both causing a rapid decalcification and acidification of the sediment (Scheffer & Schachtschabel, 2018). Covered by younger tidal flat deposits of facies type C, it represents a fossil soil horizon called "Dwogmarsch" (Müller, 1958; LLUR, 2012; Scheder et al., 2018).

Facies type D deposits were only found in vibracores RUN 26A and RUN 27A.

Facies type X consists of compact black organic mud penetrated by thick roots of reed. Sediments likely originate from a (semi-)terrestrial swampy environment. Root canals filled with tidal flat sand indicate a post-depositional exposition in the tidal flats. The unit seems to represent the base of the former dyke that was well visible in the tidal flats in the 1920s and 1930s (Busch, 1923) but became subsequently covered by sediments.

Facies type H finally marks the upper part of all the vibracores drilled and consists of recent tidal sand flat deposits today covering the archaeological remains. Its lower base is generally marked by a distinct erosive contact.

Vibracore transects A and B (Fig. 5a, b) highlight both stratigraphic similarities and changes throughout the study area and provide insights to the site's landscape dynamics. Both transects show an even distribution of basal facies type F shallow water quiet reach deposits, locally intersected by sand or shell debris of facies type G. The subsequent facies type E appears in varying depths along transect B but was only discovered to the very south of transect A (RUN 13A). Here, it is covered by c. 0.5 m of recent tidal flat sediments (facies type H) that become significantly thicker along the northern transect. Facies type H reaches a maximum thickness of c. 1.5 m at sites RUN 25A and RUN 20A. This seems to correlate with the transition from magnetic area II (RUN 13A) to I (RUN 15A, RUN 20A). At site 25A, recent deposits lie directly on top facies type F (RUN 25A), while at site RUN 20A, they cover the just locally appearing facies type X in a same stratigraphic position. Facies type D occurs only along transect B, extending from Hallig Südfall (RUN 17A, Hadler et al. submitted) towards the study area. While the unit is well preserved in the Südfall stratigraphy (RUN 17A), it is significantly thinner at coring site RUN 27A. At site RUN 26A, deposits are again well preserved but lie some decimeters deeper. Unit D is absent at site RUN 13A. Like in transect A, unit H covers the underlying units at all coring sites of transect B with a

distinct erosive contact.

Reflection Seismics

The profiles of seismic data displayed in Fig. 3 were conducted based on the magnetic gradiometry results to get vertical crossections of the magnetic anomalies. Beyond that, seismics allowed to track and extrapolate the observed structures, where magnetic gradiometry cannot go. As an excerpt of the seismic results, we show example profiles that comprise the basic magnetic features listed above (Fig. 6) and then examples on that we were able to perform corings to understand the observed stratigraphy (Fig. 7).

Fig. 6 shows five example profiles crossing the magnetic anomalies that were labeled in Fig. 4. Fig. 6 a) crosses feature IV (marked in blue here) and the transition between areas I and II (marked in white), as well as feature VI. Feature IV is only covered half and is spatially connected to a south dipping reflector at 1 m to 3 m below seafloor. The transition between I and II corresponds to the beginning of a north dipping reflector in the depth range of 1 m below seafloor. There is no reflection event that corresponds to feature IV. Fig. 6 b) again crosses feature IV and both transitions between I and II. Feature IV now is connected to a depression shaped reflection event starting at about 1 m below seafloor. Also, area I corresponds to a shallow but large depression in the first meter below seafloor. Fig. 6 c) crosses feature IV in a region where it shows higher magnetic signal amplitude (area II). Again the feature is visible as a sequence of depression shaped reflectors but visible from 1 m to about 4 m below seafloor, whereas when again crossed in area I (Fig. 6 d)), the reflection feature is only visible from 1 m to about 2 m below seafloor. Fig. 6 e) crosses both feature IV and V, both relating to depression shaped but connected seismic reflection events.

Fig 6. Results seismic examples a)-e) example seismic profiles and down left a map, showing their locations. Blue and white rectangles (profiles) and lines (map) indicate the position of several striking features in the magnetic map and their corresponding features in the seismic profiles.

In Fig. 7 we combine vibracore stratigraphies with seismic results. Fig. 7 lower right shows a map of the seismic profile section and the positions of the corresponding coring sites. Fig. 7 a) offers a complete cross section of the depression shaped reflector in the first meter below seafloor, which corresponds to the area of weak magnetic amplitude (area I in Fig. 4). Core 27A reveals that this top layer can be identified as recent tidal sand flat (facies type H), making the depression and its inner sequence of north dipping reflectors a former tidal creek that was moving northward. The same accounts for the top layer of Fig. 7 b) and core 26A. Fig. 7 a) highlights also seismic reflections that correspond to deposits of facies type D and E, that thin towards the center of the tidal creek. Due to the minor thickness of facies type D, there is no visible contrast to facies type E. The northward dipping reflector at the base of the recent tidal flat deposits marks the erosive contact between facies type H and underlying facies types. The reflector's dipping and disappearance of facies types D and E in the northern part of the profile both emphasize the erosion of older sediments by the tidal creek. The lagoonal deposits of facies type E are underlain by similar sediments of facies type F that rather reflect different ecological than deposition conditions, so accordingly the interface alone shows a small impedance contrast in the seismic section. The lagoonal shallow water quiet reach environment of facies type F is intersected by a sequence of shell and sand layers visible as reverberating seismic events. At the deepest part of the profile, high amplitude reflectors at different depths and extents are visible. Fig. 7 b) shows a similar stratigraphy except one feature. The uppermost part of the

depression shaped reflector at coring site RUN 26A, visible at depths of about 1 m to 4 m and pointed out in Fig. 6, corresponds to remains of fossil marsh (facies type D). Unlike in Fig. 7a, here facies type D is quite well discernible from underlying facies type E by a depression-shaped reflector. Along the profile, fossil marsh deposits only seem to be preserved within the depression - an observation that corresponds well to the facies type's lower stratigraphic position observed along vibracore transect B (Fig. 5c). In the northern section of the profile, the reflector marking the erosive contact at the base of facies type H reaches its deepest position about 2.5 m below ground surface.

Fig 7. Results seismic and coring a)-c) example seismic profiles and their preliminary interpretation based on vibracoring results, which are also indicated by coloured bars. The position of the profiles and the cores is shown in the map down right.

Fig. 7 c) completes the picture of the depression-shaped reflectors. At site RUN 20A, the deeper part of the depression can be assigned to facies type X, associated with the base of the former dyke. The seismic section also shows the extend of erosion by the tidal creek. At coring site RUN 13A, facies type E deposits are still preserved but completely eroded towards the central part of the tidal creek (RUN 25A, RUN 20A). It appears that comparable to site RUN 26A, facies type X deposits are merely preserved as a result of unit deformation and vertical dislocation into the observed depressions. As the slight upward bulge of the erosive contact indicates, facies type X withstood some erosion, likely due to the sediment's compact character.

Discussion

In this paper, we deal with the prospection of drowned medieval archaeological remains in the tidal flats of the German North Sea coast. Accross the entire North sea area, investigations on paleolandscapes that were drowned due to sealevel rise and storm or Tsunami events are manifold (see e.g. Van de Noort (2011) or Peeters (2017)). A main focus lies on prehistoric sites (see e.g. Bailey et al. (2020)) like the Mesolithic Doggerland hit by the Storrega tsunami (Walker et al., 2020). Prospecting these offshore sites usually involves diving activities in combination with preceding seismic and sidescan or multibeam sonar investigations. Besides these distinct offshore studies, only in Denmark, Germany, the Netherlands, Belgium and Great Britain, noteworthy tidal flat areas that contain archaeological features have been investigated. In Belgium for example, these studies especially involve high resolution marine seismic prospection. Missiaen et al. (2010) e.g. showed the potential of 2D/3D seismic echosounder data to image small scale wooden objects in the wadden sea environment. The investigated site belongs to the domain of Walraversijde (Belgium), were the remnants of a late Medieval settlement, both at the beach and inland were investigated. Due to severe coastal erosion, the settlement, dating from the late 13th century, was lost to the sea and relocated behind a dyke in the early 15th century. This is a different environmental setting compared to the Rungholt settlement which is constantly exposed to the wadden sea since the late 13th century until today. Missiaen et al. (2017) showed the potential of high resolution seismic measurements to image the very shallow stratigraphic layers offshore Raversijde, a site with roman and medieval remains. They also showed a combination of seismic measurements with terrestrial electromagnetic induction as well as Cone Penetration Tests (CPT) at a test area in the intertidal zone, imaging a peat excavation zone. Despite the differences in surrounding and timeframe, these works are strong examples showing the potential of high resolution marine seismic methods to image the very shallow stratigraphy as well as archaeological objects of different scales,

including wooden constructions or peat structures in tidal flat areas. In the United Kingdom, besides the investigations of Gaffney et al. (2007) dealing with the Paleolandscape of Doggerland based on industry seismic and acoustic data, an example of geophysical investigation of a North Sea coastal site is the former medieval port and town of Dunwich which was lost due to cliff erosion and investigated by multibeam-, side-scan sonar-, and singlebeam sub-bottom seismic data (Sear et al., 2011). In the Netherlands, the former tidal flats and salt marshes with remains of settlements from the Pre-Roman Iron Age to the Medieval period are today mostly situated behind the modern dykes, and thus can be investigated by conventional archaeological prospection methods and excavations (see e.g. van Popta (2019) or Bazelmans et al. (2012)). In Germany, different research approaches dealt with the cultural heritage of the wadden sea: In Lower Saxony, several survey campaigns have been performed (Goldhammer & Karle, 2015), analysing archives, aerial pictures, archaeological- and geological data. Goldhammer & Karle (2015) also showed a first result of tidal flat magnetic gradiometry, imaging a farmstead and parts of a tidal creek. In North Frisia, several archaeological surveys were performed in the past (e.g. Kühn, 2007) as well as a study on synthetic aperture radar (SAR) (Gade et al., 2017). Gade et al. (2017) showed that high-resolution space-borne SAR imagery with a resolution of one square meter can be used to complement archaeological surveys on intertidal flats. They were able to detect remains of farmhouse foundations and of former systems of ditches, dating to the 13-17th century AD. Nevertheless, like aerial photography, SAR images are constrained to exposed archaeological features although not limited to daylight. Since SAR-images can be used for large-scale surveillance and provide information on surface roughness, recurrent SAR-prospection might be a tool to monitor ongoing erosion in zones with archaeological remains, as our study demonstrates the scale of losses that can be inflicted by moving tidal creeks. For structures that are buried in the sediment, the methodological approach presented in this study for the first time gives a comprehensive picture of settlement areas of that time. The presented Niedam area is a unique application area, combining both very shallow stratigraphic imaging (including tidal creek erosional interfaces) and the remains of former archaeological structures like dykes and terps as interlinked prospection targets.

To finalize the picture of the presented study area, provided by magnetic gradiometry, reflection seismics and vibracores, a synoptic interpretation against both the archaeological record and literature is discussed in the following. The basic interpretation of the data starts with the different features in the magnetic map (Fig. 4) and comparing them to known archaeological structures, observed in the 1920s-1960s (Fig. 2). It is striking that feature IV (Fig. 4) follows the same path as the observed remains of the medieval dyke. Its exact - and due to the sedimentary cover so far unknown - course can now be precisely located based on magnetic gradiometry. Adding the seismic and coring results, the depression-shaped reflectors holding compact fossil marsh (facies type D in RUN 26A) and organic mud deposits (facies type X, RUN 20A) can also be linked to the medieval dyke system. The unit's local appearance and its stratigraphic position imply that the magnetic results do not show the body but only an imprint of the former dyke, as underlying deposits were deformed by the imposed load of the structure (e.g. the bend-down marsh deposits of facies type D visible in the seismics). The load of the dyke thus leaves an imprint of compressed marshland (facies type D in RUN 26A) or (semi-)terrestrial swampy areas (facies type X in RUN 20A) on which it was built. This effect was already observed by Busch (1936; summarized by Kühn et al. (2013)) on the exposed remains of the Niedam-dike, but can now be mapped by seismics. This approach provides a valuable tool to track former dyke lines in the Wadden Sea or on land, where all remains of the building itself are gone. The amount of deformation by the dyke may be estimated to at least 20 cm from vibracore

data (RUN 26, Fig. 5c), while seismic prospection indicates an even higher amount of deformation of c. 0.5 m (Fig. 7a).

Based on vibracore stratigraphies, we can also derive the cause for changes in magnetic amplitudes between areas I and II. Both seismic and coring results show a thickening and deepening of the recent tidal flat deposits in area I (facies type H in RUN 25A and RUN 20A), as well as sequences of dipping reflection events within this top layer. Compared to maps and aerial images of the study area (LVermSH, 1986; 2005; LVermGeo SH, 2018), we identified area I as the central channel of a tidal creek active in the 1980s, but silted-up today. The petering of the pre-marsh deposits (facies type E in RUN 13A) towards the center of the tidal creek and a thinner imprint of the dyke (lacking the top layer) attest to severe erosion by the tidal creek in area I. Compared to levellings of the marsh surface exposed in the study area in the 1920s (Busch, 1963), vibracores and seismic profiles indicate a general lowering of the medieval ground surface of ca. 0.5 m by gradual erosion. In the central part of the tidal inlet, the vertical erosion even reaches 1.5 m to 2 m below present day ground surface (Figs. 5b) and 7b). These results demonstrate the effects of tidal dynamics (e.g. in form of shifting tidal inlets) to the cultural heritage preserved in the Wadden Sea. With the top layer being eroded and a thick sediment cover on objects in greater depth, the remaining subsurface structures create a much weaker magnetic signal. Our results show that rapidly shifting tidal creeks - a common feature throughout the tidal flats - pose a relevant threat to the cultural heritage, emphasizing the need for proper prospection approaches of the medieval remains preserved in this amphibious landscape.

Besides the tidal creek and the dyke, both yielding the largest anomalies, the magnetic map and seismic profiles also show several smaller features. Regarding the two tidal gates, no evidence of any building structure was discovered in the respective seismic profiles. Wooden beams of the larger tidal gate were still visible and partly recovered in the 1960s by Busch (1963), who locates the base of the gate at -1.30 m a.s.l. Reconstructing the depth of the former tidal inlet from seismic profiles and nearby vibracoring sites RUN 20A and RUN 25A (Fig. 7c), we conclude that during the last decades, up to 2 m of sediment including the archaeological remains were eroded by the tidal creek and tidal dynamics. It must therefore be assumed that the last remains of the exceptional tidal gates are finally destroyed and became lost to the sea. There is, however, still evidence of their former location. Just south of the dyke, feature VII in the magnetic map shows two linear anomalies extending south from the gates' assumed position (based on 2) to a basin-like area of slightly negative anomaly. While the former likely reflect channel structures such as drainage ditches, the latter strongly resembles some kind of (natural) harbour basin. Busch (1923, 1963) describes the tidal gates as c. 25 m long and 40 m apart in an E-W-direction. His measurements fit quite well to magnetic as well as seismic results, where the imprint of the dyke reveals a basal width of c. 25-30 m and a distance of c. 35 m between the anomalies at the gates' assumed locations. We therefore conclude that although the archaeological remains are lost, we were able to reconstruct the former location of the tidal gates and even provide some new evidence for their connection to a harbour site (Fig. 8).

Fig 8. Interpretation. a) Final interpretation of the dataset. b) Comparison of the results in a) with old recordings (lines outlining the features shown in Figure 2).

Based on our studies, we were able to verify the general observations and locations already recorded at the beginning of the 20th century. However, there are considerable deviations from old observations regarding the exact position, but also in terms of interpretation of the individual archaeological features. An overview of the results and a comparison with old recordings is displayed in Fig. 8 a and b. At former times, surface

finds were in parts misinterpreted. Besides the position of the dyke and the tidal gates, also the distribution of terps needs to be changed based on the geophysical prospection presented by this paper. All terps visible in the magnetic map are connected to the dyke, rectangular in shape and arranged mostly perpendicular to the dyke. They are invariable behind the dyke and not part of the dyke body itself as described by Busch (1923; 1971). Furthermore, we did not find a so far presumend large but single terp complex, but instead 4 rectangular terps located close to each other.

East of this terp group we could identify a potential dyke branch-off (see position marked by a black star in Fig. 8) . The same interpretation would apply for another branch-off from the main dyke (marked by a black hexagon in Fig. 8), both possibly forming another small polder just south of the Niedam dyke (indicated by the dashed arrow in Fig. 8). The polder would fit well to another observation of fossil farmland just south of the main dyke in this area (Busch, 1923).

Comparing the depth of imprints in the seismic results , the imprint of the dyke is deeper than that of the terps associated with it. This would imply that the dyke might have been higher than the terps, questioning the interpretation that only terps were able to protect from the winter storm floods (Kühn, 1992a). Furthermore the imprint of the dyke appears symmetrical in its shape, indicating that the former dyke was symmetrical and was not built with a lower seaward slope as expected by Kühn (1989). Due to the greater height of the dyke compared to the terps behind it, the exeptionally large width of the dyke base of about 35 m and the installed and once completely replaced tidal gates, it is likely that the Niedam dke functioned as a seaward outer dyke. In the literature, however, it is considered to be an inland middle dyke. This is based primarily on the supposed observation that the terps were built on top of the dyke (Muuß, 1934; Kühn et al., 2013; Panten, 2016b). However, this is not the case according to our prospection results. A comparison with archaeological excavation results of a roughly contemporaneous dyke on the island of Nordstrand also emphasizes the presumed protective function of the Niedam dyke: on Nordstrand, a dyke with a width of about 10 m and a symmetric shape was built somewhat remote from the direct coastline in the 14th century, probably as a reaction to the 1362 flood. Only after the loss of its protective function, it was overbuilt with a dyke warft on top (Kühn & Müller-Wille, 1988; Kühn, 1989). Especially the great width assigns an active protective function to the Niedam dyke, which is not affected by the terps in its back. Furthermore, the tidal gates and the postulated harbour basin would be nonfunctional, given the dyke was a middle dyke.

Conclusion

We present geophysical and geoarchaeological investigations for an area in the North Frisian Wadden Sea holding remains of the sunken medieval settlement of Rungholt. The investigated area is representative for the medieval dyke system of the region, including harbour structures connected to tidal gates, housing and storage terps. We showed that the delineated prospection setup, using magnetic gradiometry and vibracoring during low tide and marine reflection seismics during high tide, is highly suitable for imaging and understanding the remains of the dyke system of Rungholt, its extent, as well as associated settlement structures. Our results further show the critical state of preservation and endangerment of the medieval cultural heritage by the dynamically changing tidal flat environment. Tidal gates, terps and dyke structures, still to be seen in the 1920s, are partly affected by erosion of up to two meters. Based on geophysical and geoarchaeological data, we showed that the imprint of former coastal protection measures on the underlying sediments is still detectable and gives a clear picture of the dyke system, although the associated archaeological remains have already

disappeared. Moreover, we detected that terps are largely rectangular and not part of the dyke body itself but attached to it. We found that these terps were probably not higher than the dyke body making the dyke more important for protection against winter storms. Finally, we showed that the crosssection of the dyke itself was of symmetrical shape and not asymmetrical as previously thought. The presented work is highly relevant for future geophysical and geoarchaeological prospection in tidal flat areas and a significant base for the reconstruction of passed cultural landscapes. The presented approach enables to investigate cultural remains and their state of preservation in a landscape of exceptional archaeological value.

Acknowledgments

This study was carried out within the framework of the DFG priority program 1630 "Harbours from the Roman Periods to the Middle Ages" (RA 496/26-2, ZI 721/10-2, ET 20/7-2, HA 7647/1-1, VO 938/21-1) and the ROOTS Cluster of Excellence funded by the German Research Foundation (EXC 2150–390870439). The authors would like to thank Detlef Schulte-Kortnack and Clemens Mohr for their extensive technical help, and Gunda and Gonne Erichsen for their logistic support on Hallig Südfall. We would also like to thank the AXIO-NET PED-Service for providing their RTK-correction data.

References

Eckelmann W., editor. Bodenkundliche Kartieranleitung (Manual of soil mapping (KA5)), 5th Ed. Stuttgart: Schweizerbart; 2005. ISBN 978-3-510-95920-4

Århammar N. Zur Vor- und Frühgeschichte der Nordfriesen und des Nordfriesischen. In: Faltings VF, Walker AGH, and Wilts, O, Editors. Friesische Studien II. Beiträge des Föhrer Symposiums zur Friesischen Philologie vom 7.-8. April 1994. Odense; 1995

Bailey G, Galanidou N, Peeters H, Jöns H, and Mennenga M, editors. The Archaeology of Europe's Drowned Landscapes. Cham:Springer; 2020. ISBN 978-3-030-37366-5

Bailey G, Harff J, and Sakellariou D, editors. Under the Sea: Archaeology and Palaeolandscapes. Cham:Springer; 2017. ISBN 978-3-319-53160-1

Bantelmann A. Die Landschaftsentwicklung an der schleswig-holsteinischen Westküste, dargestellt am Beispiel Nordfriesland. Eine Funktionschronik durch fünf Jahrtausende. Die Küste 14/2; 1966. p. 5-99. https://izw.baw.de/publikationen/die-kueste/0/k014-21966.pdf

Barmeyer H. Die Sielhafenorte in der oldenburgisch-ostfriesischen Küstenmarsch zwischen Weser und Ems. Deutsches Schiffahrtsarchiv 1975; (1)11-23. German

Bartholdy J. The backbarrier sediments of the Skallingen Peninsula, Denmark. Geografisk Tidsskrift-Danish Journal of Geography 1997; 97(1): 11-32.

Bartholdy J. Salt marsh sedimentation. In: Davis Jr RA and Dalrymple RW, editors. Principles of tidal sedimentology. Dordrecht:Springer; 2012. pp. 151-185.

Bazelmans J, Meier D, Nieuwhof A, Spek T, Vos P. Understanding the cultural historical value of the Wadden Sea region. The co-evolution of environment and society in the Wadden Sea area in the Holocene up until early modern times (11,700 BCe1800 AD): An outline. Ocean Coastal Management 2012; 68:114-126

Beeftink WG and Rozema J. The nature and functioning of salt marshes. In: Salomons W, Bayne BL, Duursma EK and Förstner U, editors. Pollution of the North Sea. Berlin: Springer; 1993. p. 59-87.

Behre K-E. A new Holocene sea-level curve for the southern North Sea. Boreas, 2007; 36: 82-102.

Bungenstock F and Weertz HJT. The high-resolution Holocene sea-level curve for Northwest Germany: global signals, local effects or data-artifacts? Int J Earth Sci (Geol Rundschau) 2010; 99:1687-1706.

Busch A. Die Entdeckung der letzten Spuren Rungholts. Jahrbuch des nordfriesischen Vereins für Heimatkunde und Heimatliebe 1923;10:3-32. German

Busch A. Neue Beobachtungen im Rungholt-Watt im Jahre 1935. Die Heimat 1936; 46(3):71-79. https://resolver.sub.uni-hamburg.de/kitodo/PPN846060221$_0$046.*German*

Busch A. Zur Rekonstruktion der Rungholter Schleusen. Die Heimat 1963; 70(6):163-168. https://resolver.sub.uni-hamburg.de/kitodo/PPN846060221$_0$070.*German*

Busch A. Wo lag vor 1200 die Schiffsanlegestelle von Grote Rungholt, und warum wurde sie nach Lütke Rungholt verlegt? Die Heimat 1964; 71(3):74-78. https://resolver.sub.uni-hamburg.de/kitodo/PPN846060221$_0$071.*German*

Busch A. 50 Jahre Rungholtforschung. Die Heimat 1971;78(6): 153-160. https://resolver.sub.uni-hamburg.de/kitodo/PPN846060221$_0$078.*German*

Carmiggelt A, Guiran T. Archeologie en historie van de sluizen in de Rotte-Dam. Rotterdams Jaarboekje 2002; 10(10):166-176. Dutch

Gade M, Kohlus J, and Kost C. SAR Imaging of Archaeological Sites on Intertidal Flats in the German Wadden Sea. Geosciences 2017; 7:105. doi:10.3390/geosciences7040105

Gaffney V, Thomson K, Finch S. Mapping Doggerland: The Mesolithic Landscapes of the Southern North Sea. Archaeopress; 2007. ISBN-13 : 978-1905739141

Walker J, Gaffney V, Fitch S, Muru M, Fraser A, Bates M, Bates R. A great wave: the Storegga tsunami and the end of Doggerland?. Antiquity 2020; 94(378):1409-1425.

Goldhammer J, Karle M. Geoarchäologie im Schlick: Das Projekt *Besiedlungs- und Kulturgeschichte des niedersächsischen Wattenmeerraumes.* Coastline Reports 2015; (25):1-9. ISSN 0928-2734, ISBN 978-3-939206-18-7. German.

Grøn O, Boldreel LO. Sub-bottom profiling for large-scale maritime archaeological survey. Proceedings of the 2013 MTS/IEEE OCEANS; 2013 June 10–14; Bergen, Norway.doi: 10.1109/OCEANS-Bergen.2013.6608027

Hadler H, Vött A, Newig J, Emde K, Finkler C, Fischer P, Willershäuser T. Geoarchaeological evidence of marshland destruction in the area of Rungholt, present-day Wadden Sea around Hallig Südfall (North Frisia, Germany), by the Grote Mandrenke in 1362 AD. Quaternary International 2018a; 473:37-54.

Hadler H, Fediuk A, Fischer P, Rabbel W, Schwardt M, Wilken D, Willershäuser T, Wunderlich T, Vött A. Drowned by the Grote Mandrenke in 1362 AD – new geoarchaeological research on the late medieval trading centre Rungholt (North Frisia). In: Egberts, L., Schroor, M., editors. Waddenland Outstanding – History, Landscape and Cultural Heritage of the Wadden Sea Region. Amsterdam University Press, Amsterdam; 2018b. p. 239-252.

Hadler H, Vött A, Willershäuser T, Wilken D, Blankenfeldt R, Von Carnap-Bornheim C, Emde K, Fischer P, Ickerodt U, Klooß S, Majchczack B, Obrocki L, Rabbel W. From marshland to tidal flats – Deciphering major landscape changes and storm surge impacts around the medieval settlement of Rungholt (Wadden Sea of North Frisia, Germany) using Direct Push sensing data. Submitted to ESPL Special Issue, 2021.

Harck O. Landschaftsgeschichte und Archäologie an der Westküste der jütischen Halbinsel. In: Kossack G, Harck O, Newig J, Hoffmann D, Willkomm H, Averdieck F-R and Reichstein J. Archsum auf Sylt Teil 1. Einführung in Forschungsverlauf und Landschaftsgeschichte. Studien zur Küstenarchäologie Schleswig-Holsteins B. 1. Römisch-Germanische Forschungen 39. Mainz; 1980. p. 32-63. German

Hoffmann D. Das Küstenholozän im Einzugsbereich der Norderhever, Nordfriesland. In: Higelke B, Hoffmann D, Kühn HJ, and Müller-Wille M, editors. Norderheverprojekt 1. Landschaftsentwicklung und Siedlungsgeschichte im Einzugsgebiet der Norderhever (Nordfriesland). Offa-Bücher 66. Neumünster; 1988. p. 51-115. German.

Hoffmann D. Holocene landscape development in the marshes of the West Coast of Schleswig-Holstein, Germany. Quaternary International 2004; 112:29-36.

Kühn HJ. Deiche des Mittelalters und der frühen Neuzeit in Nordfriesland. In: Kühn HJ, Panten A, editors. Der frühe Deichbau in Nordfriesland. Verlag Nordfriisk Instituut: Bredstedt; 1995. p. 9-57. ISBN 3-88007-158-6. German

Kühn HJ. Sieben Thesen zur Frühgeschichte des Deichbaus in Nordfriesland. In: Steensen T, editor. Deichbau und Strurmfluten in den Frieslanden. Beiträge vom 2. Historiker-Treffen des Nordfriisk Instituut. Bredstedt; 1992a. p. 9-12. ISBN 3-88007-187X. German

Kühn HJ. Die Anfänge des Deichbaus in Schleswig-Holstein. Kleine Schleswig-Holstein-Bücher 42. Heide; 1992b. ISBN 3-8042-0561-5. German.

Kühn HJ. Jenseits der Deiche. Archäologie im nordfriesischen Wattenmeer. In: V. Carnap-Bornheim C, Radtke C, editors, Es war einmal ein Schiff. Hamburg:

Marebuchverlag; 2007. ISBN 978-3-86648-053-7. German.

Kühn HJ. Die Warften des 'Acht-Warften-Gebietes'. In Newig, J., Haupenthal, U. (eds.), Rungholt. Rätselhaft und widersprüchlich. Husum, Schleswig-Holstein: Husum Druck- und Verlagsgesellschaft mbH u. Co. KG; 2016. p. 56-59. ISBN 978-3-89876-824-5. German.

Kühn HJ, Müller-Wille M. Siedlungsarchäologische Untersuchungen im nordfriesischen Marschen- und Wattengebiet und in Eiderstedt. In: Higelke B, Hoffmann D, Kühn HJ, and Müller-Wille M, editors. Norderheverprojekt 1. Landschaftsentwicklung und Siedlungsgeschichte im Einzugsgebiet der Norderhever (Nordfriesland). Offa-Bücher 66. Neumünster; 1988. p. 181-193. German.

Kühn HJ, Meier D, Mertens C, Brauer R. Kulturspuren im nordfriesischen Wattenmeer. In: Meier D, Kühn HJ, Borger GJ, editors. Der Küstenatlas. Das schleswig-holsteinische Wattenmeer in Vergangenheit und Gegenwart. Heide: Boyens; 2013. p. 118-177. ISBN 978-3-8042-1381-4. German.

LVermGeo SH Landesamt für Vermessung und Geoinformation Schleswig-Holstein, 2018: ATKIS-Digitale Orthophotos DOP 20 Maßstab 1:5.000 Gebiet Südfall. Kiel. LVermLKN SH Landesbetrieb für Küstenschutz, Nationalpark und Meeresschutz

Schleswig-Holstein, 2018: Digitale Orthophotos DOP 20 Maßstab 1:5.000 Gebiet Südfall. Tönning. LVermSH Landesvermessungsamt Schleswig-Holstein, 1986: Topographische

Karte 1:25.000 Blatt 1518 Südfall. Kiel.

LVermSH Landesvermessungsamt Schleswig-Holstein, 2005: Topographische Karte 1:25.000 Blatt 1518 Südfall. Kiel. ISBN 3-89130-053-0

LLUR Landesamt für Landwirtschaft, Umwelt und ländliche Räume des Landes Schleswig Holstein, 2012. Die Böden Schleswig-Holsteins. Schriftenreihe LLUR SH – GB 23. Available from: https://www.umweltdaten.landsh.de/nuis/upool/gesamt/geologie/bodenbroschuere2019.pdf [Accessed 29 October 2020].

Meier D. Man and environment in the marsh area of Schleswig–Holstein from Roman until late Medieval times. Quaternary International 2004; 112:55-69.

Meier D. Die nordfriesischen Uthlande. In: Meier D, Kühn HJ, Borger GJ, editors. Der Küstenatlas. Das schleswig-holsteinische Wattenmeer in Vergangenheit und Gegenwart. Heide: Boyens; 2013. p. 74-117. ISBN 978-3-8042-1381-4. German.

Menke B. Die holozäne Nordseetransgression im Küstenbereich der südöstlichen deutschen Bucht. In: Higelke B, Hoffmann D, Kühn HJ, and Müller-Wille M, editors. Norderheverprojekt 1. Landschaftsentwicklung und Siedlungsgeschichte im Einzugsgebiet der Norderhever (Nordfriesland). Offa-Bücher 66. Neumünster;1988. p. 117-137. German.

Milkereit B, Spencer C. Noise suppression and coherency enhancement of seismic

data. In: Agterberg FP, Bonham[U+2010]Carter GF, editors. Statistical application in the earth sciences. 89(9). Ottawa: Geological Survey of Canada; 1989. p. 243–248.

Missiaen T. The potential of seismic imaging in marine archaeological site investigations. Relicta - Archeologie monumenten- en landschapsonderzoek in Vlaanderen / Heritage research in Flanders 6; 2010. p. 219-236

Missian T, Evangelinos D, Clearhout C, De Clerq M, Pieters M, Demerre I. Archaeological prospection of the nearshore and intertidal area using ultra-high resolution marine acoustic techniques: Results from a test study on the Belgian coast at Ostend-Raversijde. Geoarchaeology 2017:1-15. DOI: 10.1002/gea.21656

Müller W. Grundsätzliche Betrachtungen zur systematischen Gliederung der Marschböden. Geologisches Jahrbuch 76. Stuttgart: Schweizerbart; 1958. p. 11-24. German.

Muuß R. Rungholt - Ruinen unter der Friesenhallig. Franz Westphal:Lübeck; 1934. German.

Newig J. Nordfrieslands Küste zwischen Landgewinn und Landverlust. Geographische Rundschau 2014; 66(3):4-13. German. Panten A. Im salzen Meere

vergangen - Aufzeichnungen über die Flut von 1362. Nordfriesland 2012; 177:11-16. German.

Panten A. Der Reichtum der Rungholter: Sage oder Wirklichkeit? In: Newig J, Haupenthal U, editors. Rungholt. Rätselhaft und widersprüchlich. Husum Druck und Verlagsgesellschaft: Husum; 2016a. p. 41-45. ISBN 978-3-89876-824-5. German.

Panten A. Der Untergang Rungholts und des Rungholt-Koogs durch die Erste Grote Mandränke. In: Newig J, Haupenthal U, editors. Rungholt. Rätselhaft und widersprüchlich. Husum Druck und Verlagsgesellschaft: Husum; 2016b. p. 48-49. ISBN 978-3-89876-824-5. German.

Van de Noort R. North Sea Archaeologies: a maritime biography, 10,000 BC—AD 1500. Oxford University Press: Osford; 2011. ISBN 978–0–19–956620–4.

Peeters H. Review article: Coastal archaeologies: settlement on the changing North Sea littoral. Antiquity 2017; 91:358. Popta YT. No Country for Men: Searching for

Late Medieval Submerged Settlements in the North-Eastern Zuiderzee Area in the Netherlands. European Journal of Archaeology 2019; 22(4):567–587.

Quinn R, Breen C, Forsythe W. Integrated Geophysical Surveys of the French Frigate La Surveillante (1797), Bantry Bay, Co. Cork, Ireland. J. Archaeol. Sci.; 2002; 29: 413–422.

Reineck HE. Das Watt. Ablagerungs- und Lebensraum. Frankfurt/Main W. Kramer Verlag;1982. German.

Scheder J, Engel M, Bungenstock F, Pint A, Siegmüller A, Schwank S, Brückner H. Fossil bog soils (‘dwog horizons’) and their relation to Holocene coastal changes in the Jade Weser region, southern North Sea, Germany. Journal of Coastal Conservation 2018; 22:51-69.

Scheffer F and Schachtschabel P. Lehrbuch der Bodenkunde. Heidelberg: Spektrum Akademischer Verlag; 2018. German.

Schroeder D and Brümmer G. Zur Genese und Klassifizierung der Marschen. Mitteilungen der Deutschen Bodenkundlichen Gesellschaft 1968; 8:243-245. German.

Schrott L. Gelände-Arbeitsmethoden in der Geomorphologie. In: Ahnert F, editor. Einführung in die Geomorphologie. Stuttgart: UTB; 2015. p. 396–413. German.

Sear DA, Bacon SR, Murdock A, Doneghan G, Baggaley P, Serra C, LeBas TP. Cartographic, Geophysical and Diver Surveys of the Medieval Town Site at Dunwich, Suffolk, England. The International Journal of Nautical Archaeology 2011; 40(1):113–132. doi: 10.1111/j.1095-9270.2010.00275.x

Stolt RH. Migration by Fourier Transform. Geophysics 1978; 43(1):23-48

Van de Noort, R. North Sea Archaeologies: A Maritime Biography, 10,000 BC to AD 1500. Oxford University Press: Oxford; 2011. ISBN 978–0–19–956620–4

von Carnap[U+2010]Bornheim C, Kalmring S. DFG[U+2010] Schwerpunktprogramm 1630 ”Häfen von der Römischen Kaiserzeit bis zum Mittelalter. Zur Archäologie und Geschichte regionaler und überregionaler Verkehrssysteme”. Schleswig: Annual Report Centre for Baltic and Scandinavian Archaeology 2011. p. 28–31. German.

Vollmer M, Guldberg M, Maluck M, Marrewijk D, Schlicksbier G. Landscape and Cultural Heritage in the Wadden Sea Region – Project Report. Wadden Sea Ecosystem No. 12. Common Wadden Sea Secretariat: Wilhelmshaven; 2001. German.

Westley K, Mcneary R. Archaeological Applications of Low-Cost Integrated Sidescan Sonar/Single-Beam Echosounder Systems in Irish Inland Waterways. J. Archaeol. Prospect. 2017; 24:37-57.

Wilken D, Wunderlich T, Feldens P, Heinrich C. Application of 2D Fourier filtering for elimination of stripe noise in side-scan sonar mosaics. Geo-Marine Letters , 2012; 32(4):1-11. DOI: 10.1007/s00367-012-0293-z

Wilken D, Wunderlich T, Hollmann H, Schwardt M, Rabbel W, Mohr C, Schulte-Kortnack D, Nakoinz O, Enzmann J, Jürgens F, Wilkes F. Imaging a medieval shipwreck with the new PingPong 3D marine reflection seismic system. Archaeological Prospection 2019a;26: 211– 223. DOI: 10.1002/arp.1735.

Wilken D, Wunderlich T, Feldens P, Coolen J, Preston J, Mehler N. Investigating the Norse Harbour of Igaliku (Southern Greenland) Using an Integrated System of Side-Scan Sonar and High-Resolution Reflection Seismics, Remote Sens. 2019b; 11:

1889. doi:10.3390/rs11161889 742

Figure 1 Click here to access/download;Figure;FIG1-map.tif

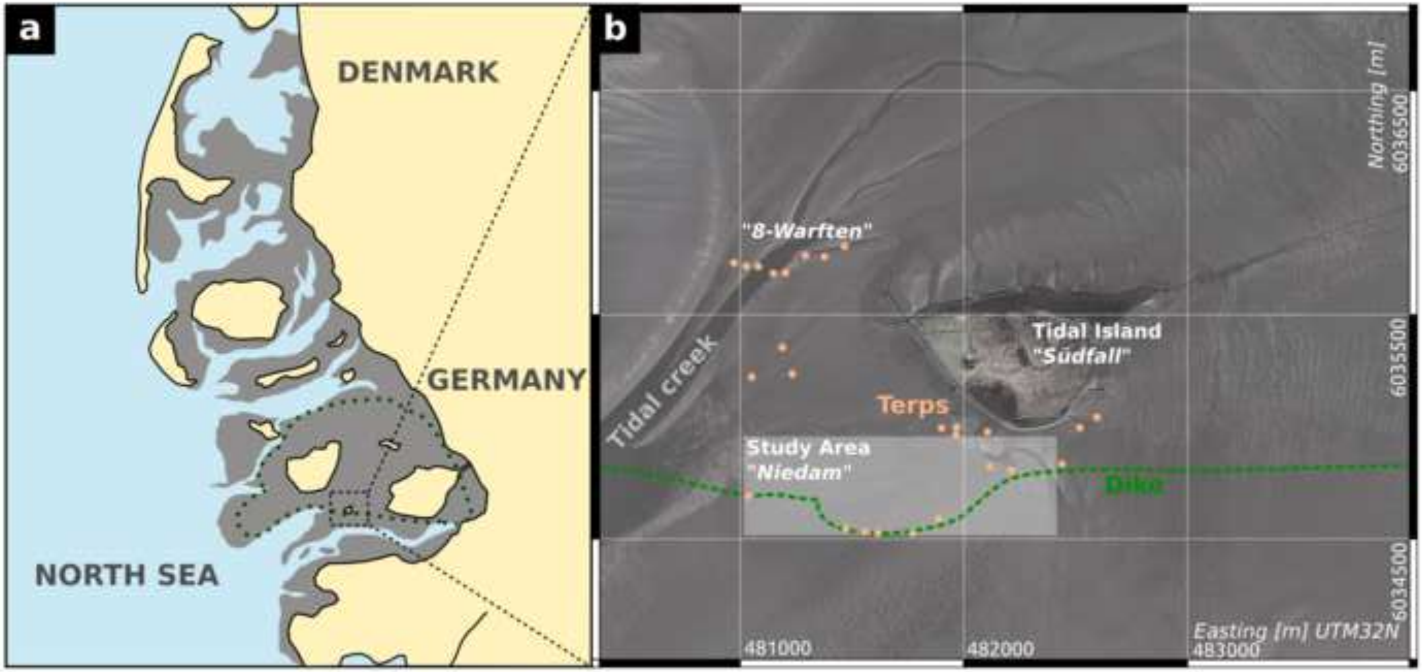

Figure 2 Click here to access/download;Figure;FIG2-dykeFinds.tif

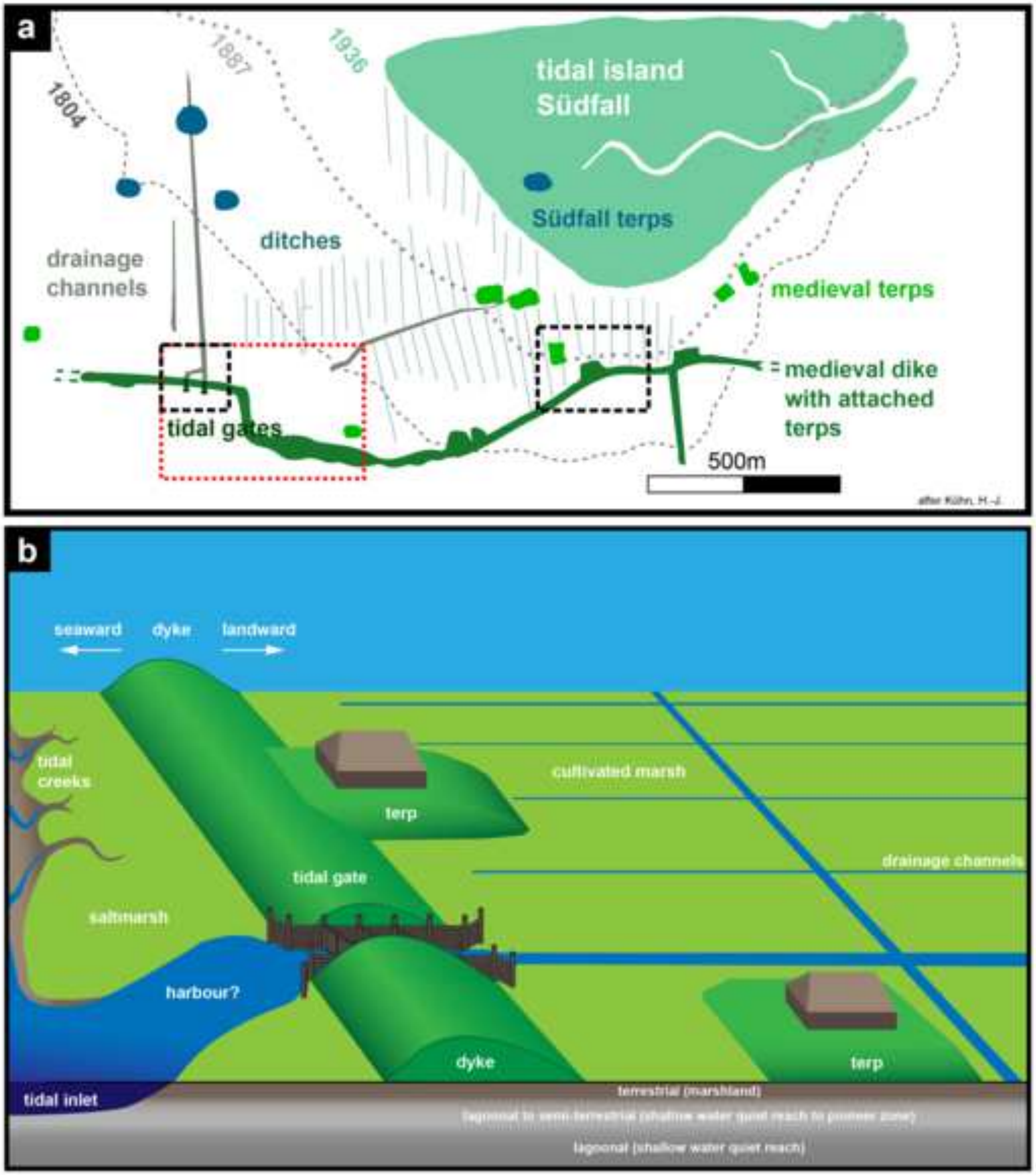

Figure 3

Click here to access/download;Figure;FIG3-methods.tif

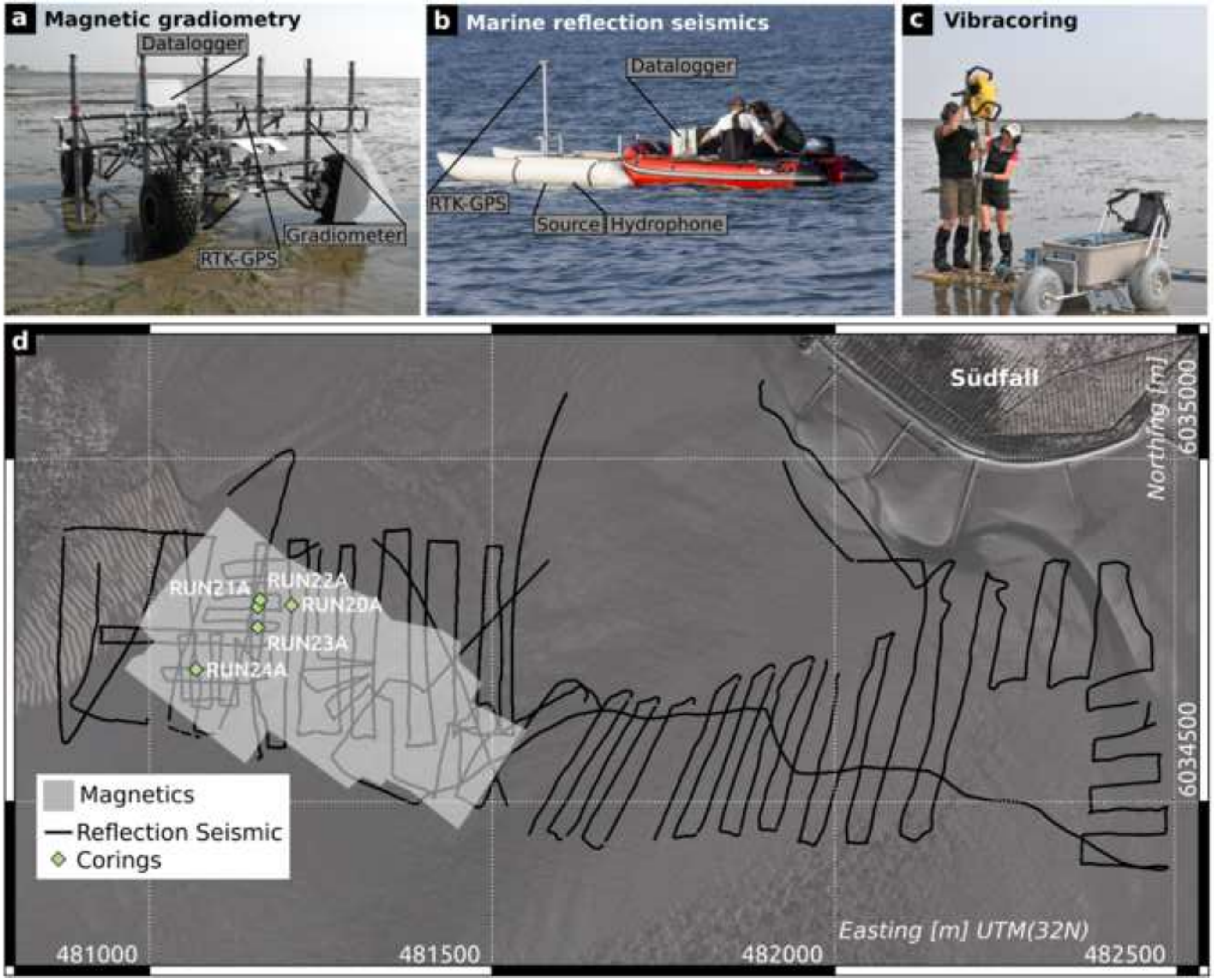

Click here to access/download;Figure;FIG4-magnetics.tif

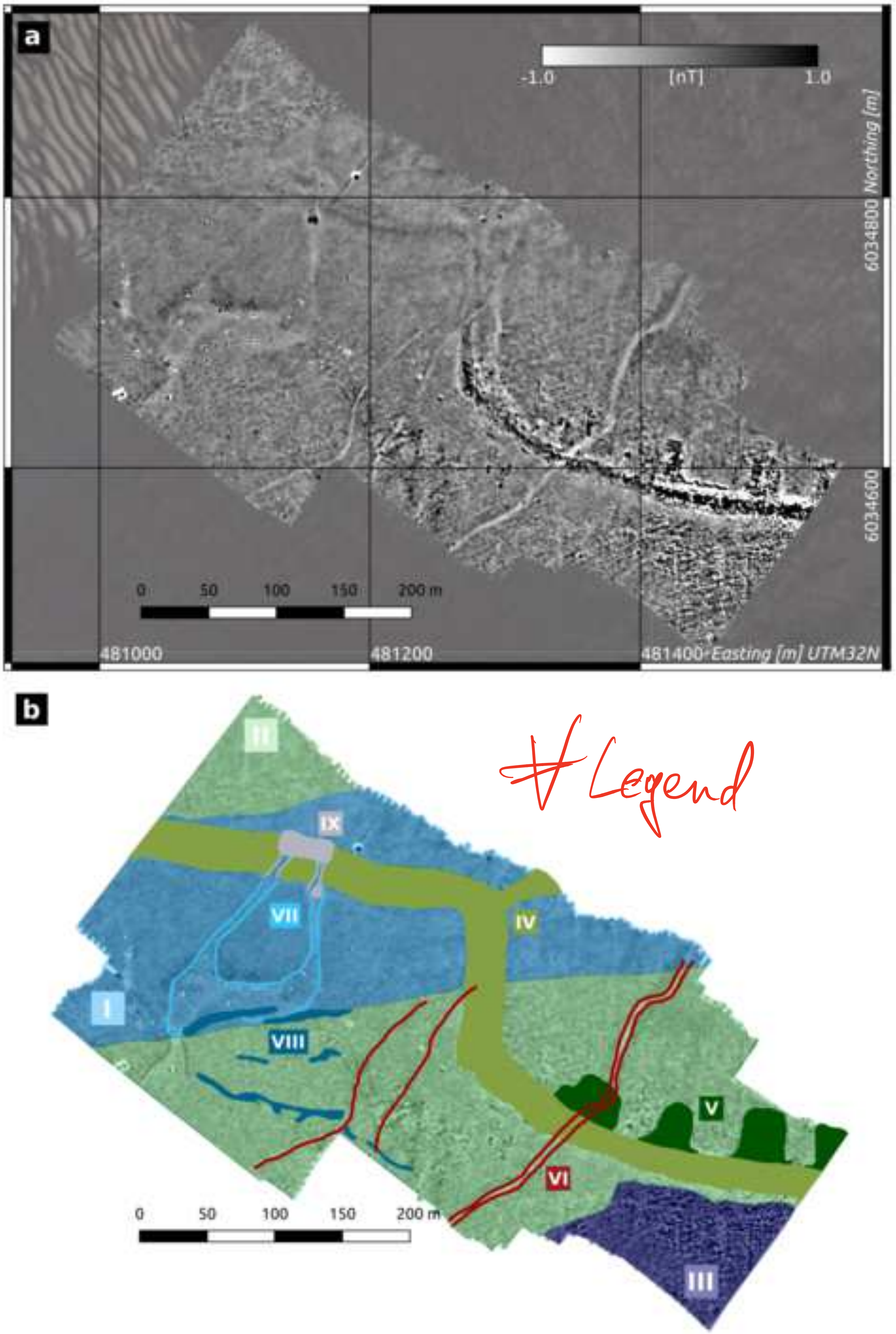

Figure 5

Click here to access/download;Figure;FIG5-transects.tif

It is unclear where the coring locations were.

Plot coring locations on magnetic map, but not the cores.

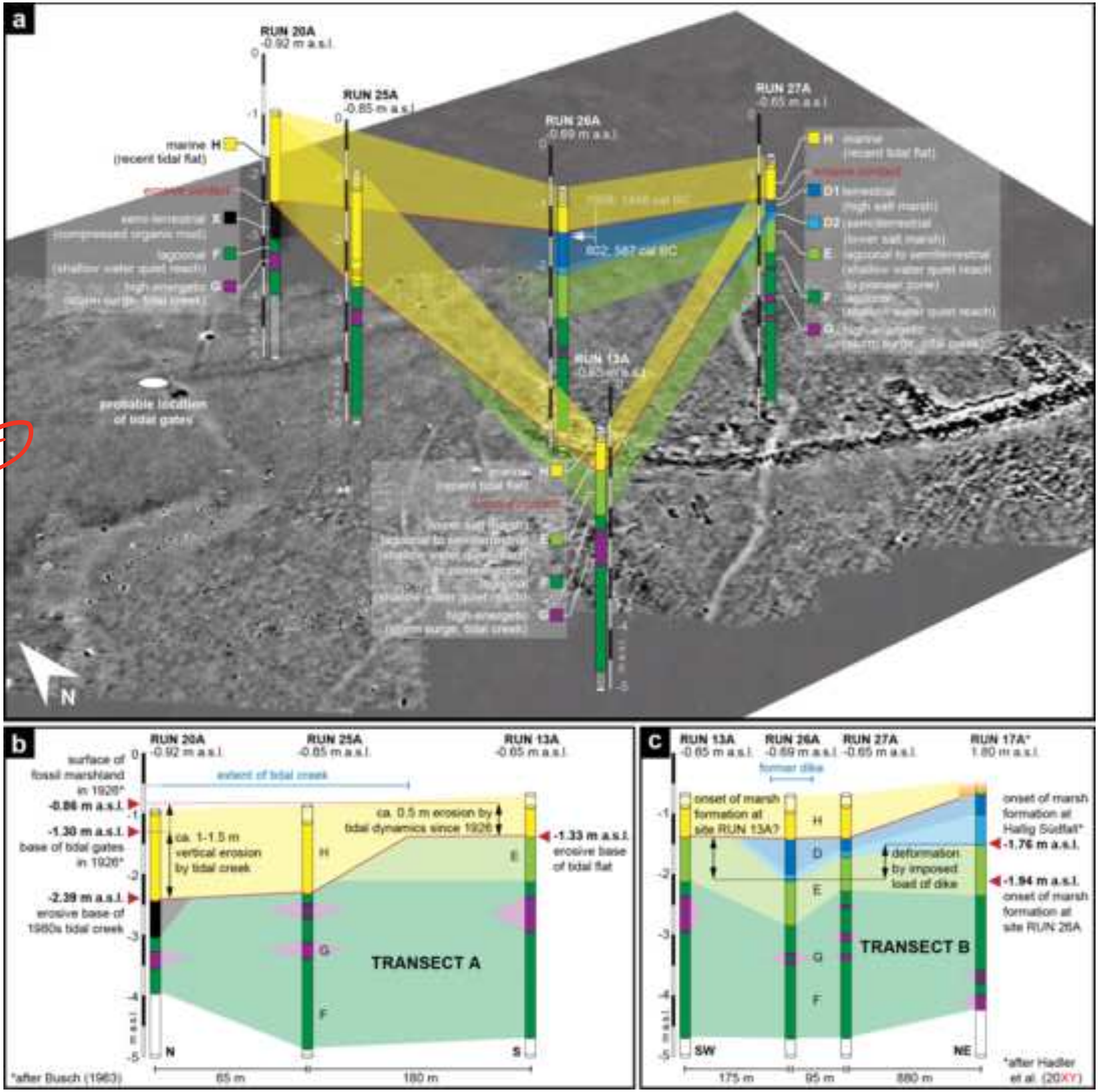

Figure 6

Click here to access/download;Figure;FIG6-seismics.tif

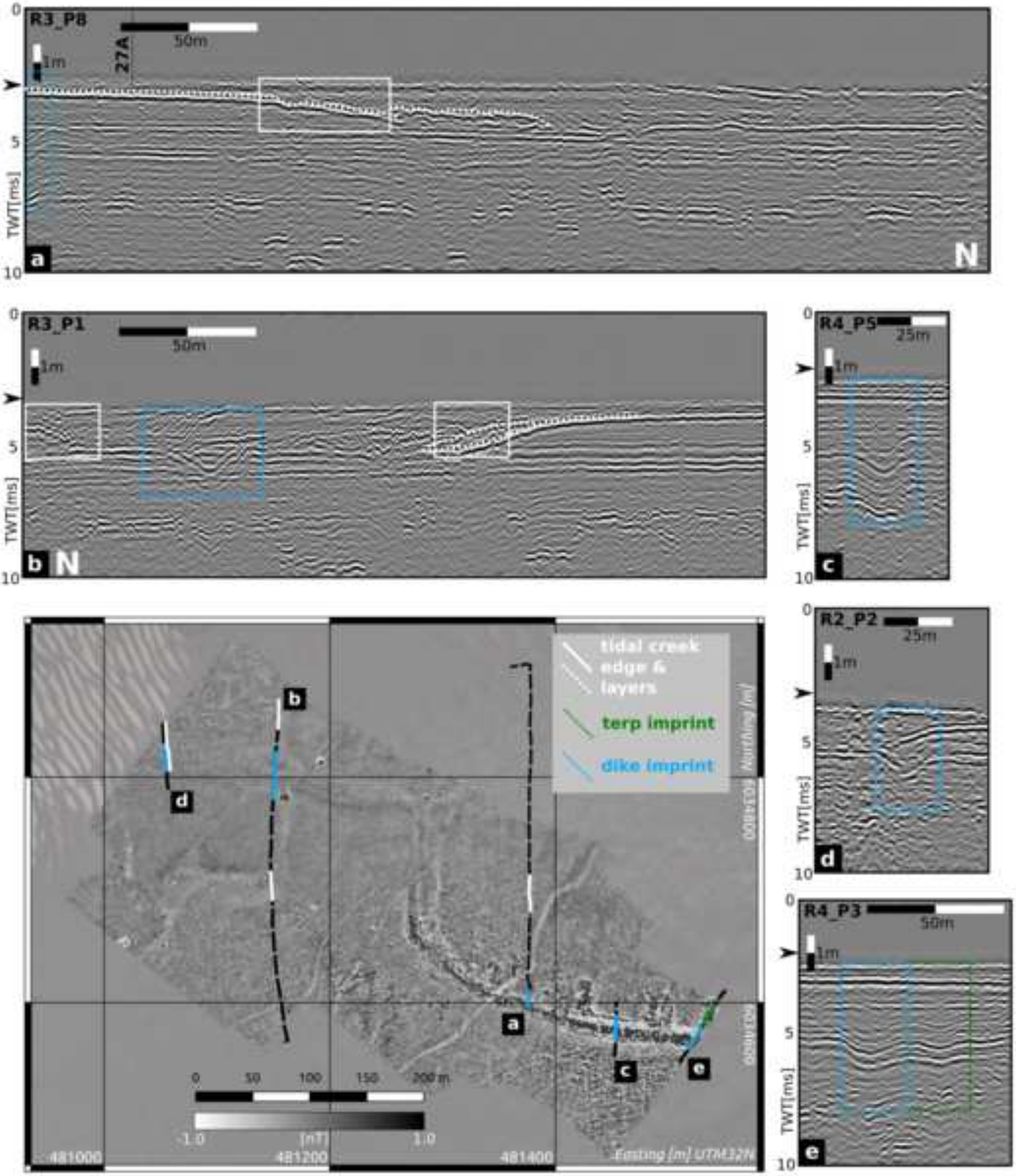

Figure 7

Click here to access/download;Figure;FIG7-interpretation1.tif

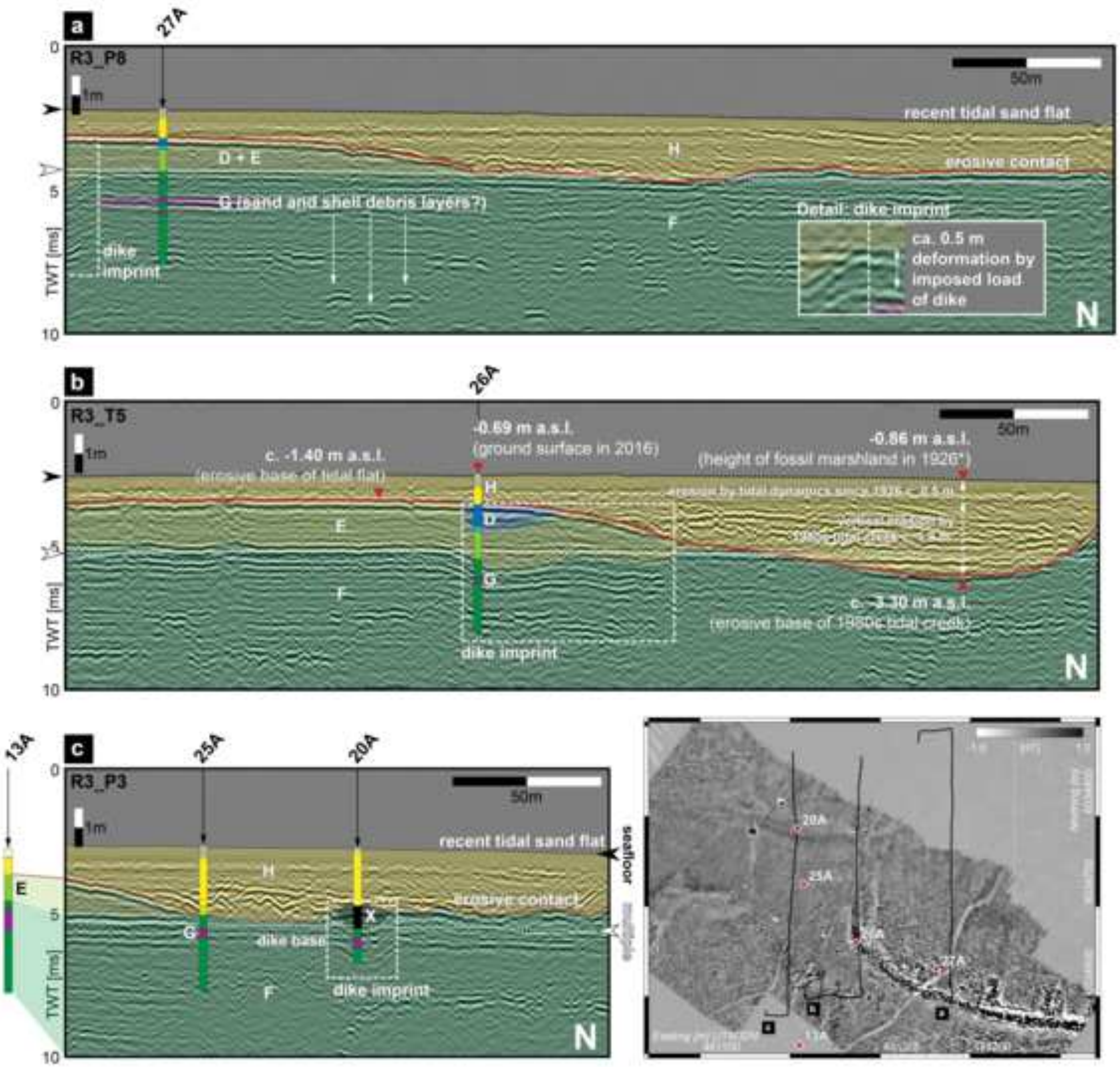

Figure 8

Click here to access/download;Figure;FIG8-interpretation2.tif

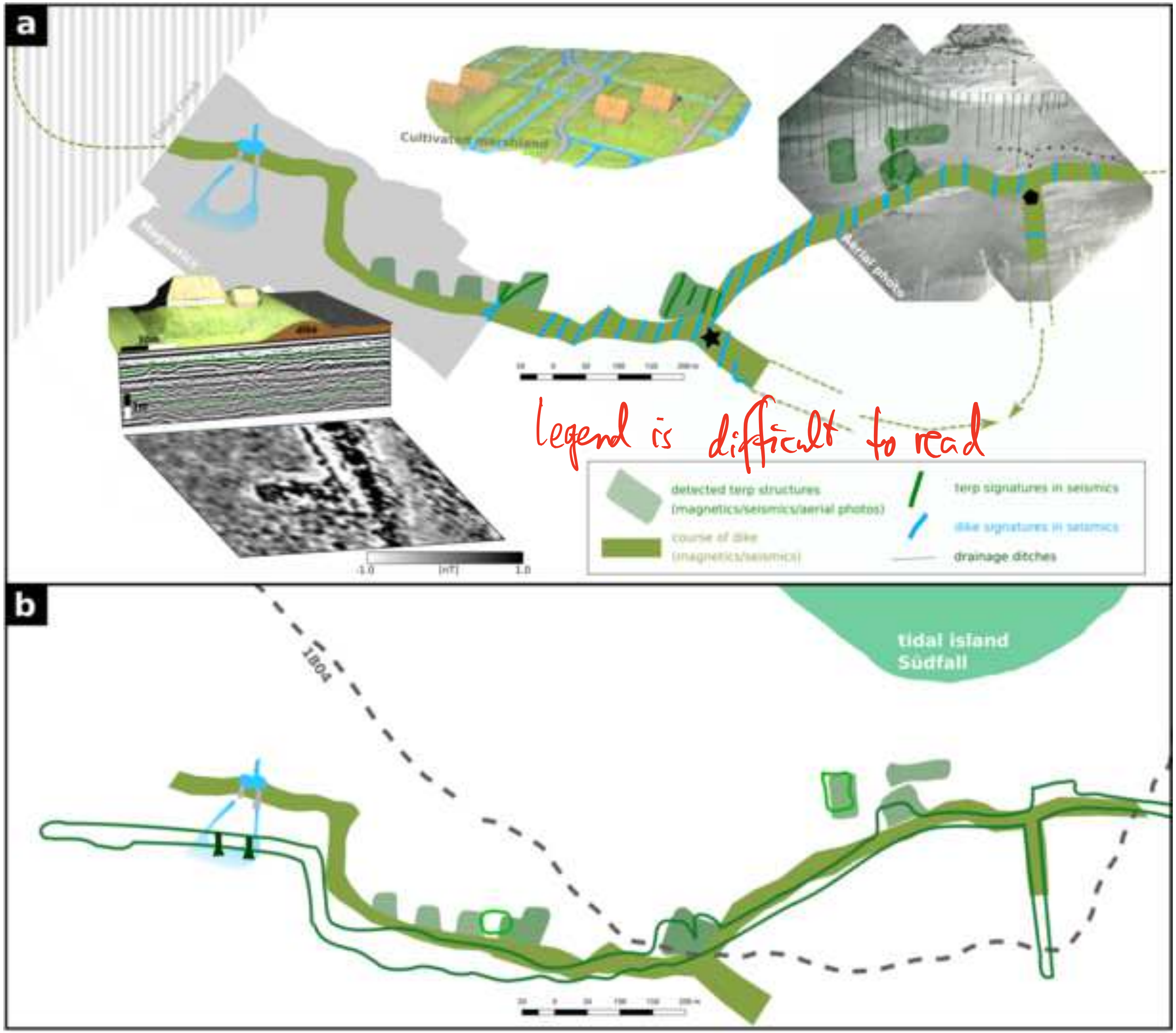

Supplement: Attachment — Submitted filename: PONE-D-21-06483_reviewer_comments.pdf [file pone.0265463.s001.pdf]
